# Administration of Ascorbic Acid Alleviates Neuronal Damage After Cerebral Ischemia in ODS Rats

**DOI:** 10.3390/antiox14070773

**Published:** 2025-06-23

**Authors:** Naohiro Iwata, Naoto Ogawa, Tom Imai, Siti Sabirah Binti Ridzuan, Shinya Kamiuchi, Hirokazu Matsuzaki, Meiyan Xuan, Bo Yuan, Mari Okazaki, Yasuhide Hibino

**Affiliations:** 1 Laboratory of Immunobiochemistry, Faculty of Pharmaceutical Sciences, Josai University, Saitama 350-0295, Japan; n-iwata@josai.ac.jp (N.I.); yv11016kuma@gmail.com (N.O.); timai@josai.ac.jp (T.I.); sabirahmsu@gmail.com (S.S.B.R.); kamiuchi@josai.ac.jp (S.K.); genbien@josai.ac.jp (M.X.); seitaib@josai.ac.jp (Y.H.); 2 Laboratory of Pharmacology, Faculty of Pharmaceutical Sciences, Josai University, Saitama 350-0295, Japan; ma-tsu@josai.ac.jp (H.M.); yuanbo@josai.ac.jp (B.Y.); 3 Laboratory of Organic and Medicinal Chemistry, Faculty of Pharmaceutical Sciences, Josai University, Saitama 350-0295, Japan

**Keywords:** L-ascorbic acid (AA), osteogenic disorder Shionogi (ODS) rat, middle cerebral artery occlusion and reperfusion (MCAO/Re), reactive oxygen species (ROS), neuronal apoptosis, pro-inflammatory cytokines, sodium-dependent vitamin C transporter 2 (SVCT2)

## Abstract

Reactive oxygen species (ROS) contribute to cerebral damage in transient cerebral ischemia, making their elimination a key therapeutic target. Osteogenic disorder Shionogi (ODS) rats, which lack endogenous L-ascorbic acid (AA) synthesis, serve as a useful model for investigating AA’s protective effects against ischemic brain injury. ODS rats were given an AA-free diet (0% AA), 0.1% AA, or 1% AA in drinking water for two weeks before undergoing middle cerebral artery occlusion and reperfusion (MCAO/Re). The 0% AA group exhibited pronounced damage following MCAO/Re, characterized by the induction of lipid peroxidation, O_2_^−^ production, inflammation-related gene expression, and extensive infarct formation. In contrast, the 1% AA group showed reductions in these markers, along with fewer TUNEL-positive cells and a smaller infarct volume. Notably, sodium-dependent vitamin C transporter 2 (SVCT2) expression increased in both two AA-supplemented groups, although the 0.1% AA group did not exhibit sufficient improvement in post-ischemic damage. A two-week intake of AA significantly alleviated MCAO/Re-mediated injuries associated with oxidative stress and inflammation in ODS rats. Sufficient AA intake is thus supposed to mitigate ischemic damage, possibly through SVCT2 upregulation and enhanced AA availability, leading to the suppression of oxidative stress and inflammation.

## 1. Introduction

L-ascorbic acid (AA) is a water-soluble vitamin with potent antioxidant activity. It plays essential physiological roles, including collagen synthesis, lipid metabolism, and functioning as a dioxygenase cofactor involved in neurotransmitter biosynthesis [1,2]. Many mammals, including rats, can biosynthesize AA from glucose in the liver, making AA deficiency a rare occurrence in these species. However, due to mutations in the gene encoding L-gulonolactone oxidase (GLO), the rate-limiting enzyme in AA synthesis, humans lack the ability to synthesize AA endogenously and must obtain it from dietary sources [3]. Although severe AA deficiency leading to scurvy is uncommon in modern societies, cross-sectional studies indicate that 10 to 15% of Western adults consume suboptimal amounts of AA, leading to chronic AA deficiency [4,5,6]. This condition is particularly prevalent in socioeconomically disadvantaged populations due to insufficient dietary intake. Additionally, certain lifestyle factors and physiological conditions—including smoking, pregnancy, excessive physical activity, aging, and metabolic syndrome-related disorders such as hypertension, diabetes mellitus, and obesity—can accelerate AA depletion, further increasing the risk of deficiency.

AA is maintained at relatively high concentrations in the central nervous system (CNS), where it plays a crucial role in protecting neurons from oxidative damage caused by reactive oxygen species (ROS) [7,8]. In cerebral ischemia, excessive ROS generation occurs due to mitochondrial electron transport chain dysfunction and arachidonic acid metabolism, leading to oxidative stress-induced neuronal damage [9,10,11]. Although previous studies have explored the cerebroprotective effects of AA in rodent models [12,13,14,15], a major limitation is that most rodents can endogenously synthesize AA. Consequently, these models fail to replicate the impact of AA deficiency, limiting their relevance to human physiology. Therefore, the use of osteogenic disorder Shionogi (ODS) rats, which lack endogenous AA synthesis, allows for a more accurate investigation of the consequences of AA deficiency in ischemic brain injury [16].

To address this issue, ODS rats, a mutant strain derived from the Wistar lineage, have been utilized as a model for AA deficiency. ODS rats lack GLO activity due to a single-base mutation in the GLO gene, which, in turn, renders them unable to synthesize AA and makes them a valuable model for studying AA deficiency similar to that observed in humans [16,17,18]. When deprived of AA for more than two weeks, ODS rats develop progressive symptoms characteristic of AA deficiency, including anorexia, weight loss, systemic hemorrhage, and gait disturbances. Prolonged AA deficiency leads to osteogenesis imperfecta, multiple fractures, and osteoporosis, ultimately resulting in mortality [16]. This phenotype closely resembles the clinical manifestations of scurvy in humans, highlighting the suitability of ODS rats for studying the pathophysiological consequences of chronic AA deficiency.

This study aims to evaluate the impact of AA deficiency on cerebral ischemia-reperfusion injury using ODS rats, a model that mimics human AA deficiency. To establish a model of chronic AA deficiency, ODS rats were maintained on an AA-free diet (0% AA) for two weeks before undergoing middle cerebral artery occlusion and reperfusion (MCAO/Re) to assess ischemic damage under AA-deficient conditions. To investigate potential AA-mediated neuroprotection, additional groups of ODS rats received drinking water containing either 0.1% AA, which reflects standard dietary intake levels, or 1% AA, representing high-dose supplementation, throughout the two-week feeding period. In addition to measuring O_2_^−^ production and lipid peroxidation levels in the cerebral cortex, we also evaluated the gene expression of inflammation-related factors and antioxidant enzymes to examine the molecular mechanisms underlying cerebroprotective effects of AA.

AA plays a critical role in maintaining redox balance in the brain, particularly during ischemic stress, where its transport and availability become crucial. Since AA cannot readily cross the blood–brain barrier (BBB), its uptake into the CNS depends on specific transporters. Sodium-dependent vitamin C transporter 2 (SVCT2) facilitates the transport of reduced AA, whereas glucose transporter 1 (GLUT1) is responsible for dehydroascorbic acid (DHA) uptake, which can subsequently be converted back to AA inside cells [19]. Under ischemic conditions, SVCT2 expression is upregulated to enhance AA transport into the CNS, promoting its neuroprotective effects by mitigating inflammatory responses associated with cerebral ischemia in rats [13,15] and mice [20]. Under ischemic conditions, increased oxidative stress converts AA into DHA, making GLUT1-mediated DHA uptake essential for maintaining intracellular AA levels [21]. Considering the roles of these transport mechanisms in regulating AA availability in the CNS, we also investigated the gene expressions of SVCT2 and GLUT1 to evaluate their potential contributions to AA transport under ischemic conditions.

## 2. Materials and Methods

### 2.1. Experimental Animals

Male osteogenic disorder Shionogi (ODS/ShiJcl-od/od) rats (7 weeks old, weighing 150–170 g) were purchased from CLEA Japan, Inc. (Tokyo, Japan) and housed under standard conditions in a temperature-controlled environment (23 ± 0.5 °C) with a 12 h light/dark cycle. During a one-week acclimation period, the rats were given ad libitum access to AA-deficient rodent chow (CL-2, CLEA Japan) and water containing 0.1% AA (L-ascorbic acid, Fujifilm Wako Chemical Co., Osaka, Japan). After acclimation, the ODS rats were divided into three groups based on their AA intake: the 0% AA group (n = 22), which received only distilled water (representing AA deficiency); the 0.1% AA group (n = 22), which received 0.1% AA in distilled water (representing standard AA supplementation, reflecting the AA content in a standard rodent diet); and the 1% AA group (n = 22), which received 1% AA in distilled water (representing high-dose AA supplementation). Sham-operated animals were not included in this study, as the primary objective was to evaluate baseline AA levels and molecular responses under non-stressed conditions. Accordingly, pre-ischemic animals (pre-MCAO/Re) served as controls. The groups were maintained under these conditions for an additional two weeks until stroke was induced by MCAO/Re. The rats exhibiting abnormalities due to AA deficiency were excluded from the experiment.

### 2.2. Middle Cerebral Artery Occlusion and Reperfusion

The experimental MCAO/Re rat model was prepared as described previously [12,13]. The rats were anesthetized with isoflurane (4% for induction and 1.5% for maintenance) (SN-487, Shinano, Tokyo, Japan) under spontaneous respiration. After a midline incision on the neck, the right common carotid artery was isolated under an operating microscope (SZX7, Olympus, Tokyo, Japan) mounted on a stand (SZ2-STU1, Olympus) to ensure precision. All branches of the external carotid artery were ligated. The tip of a 4-0 surgical nylon monofilament, rounded by flame heating, was inserted into the internal carotid artery and advanced to occlude the origin of the MCA. The rectal temperature was maintained at 37 °C using a heat lamp (TJA-550, AS ONE, Osaka, Japan) and a heating pad (BWT-100A, Bio Research Center, Nagoya, Japan) throughout the operation. After 2 h of occlusion, the filament was withdrawn to allow reperfusion. The distance from the bifurcation of the common carotid artery to the tip of the suture was approximately 20 mm in all rats. Cerebral blood flow was monitored using Laser Doppler flowmetry (ATBF-LC1, Unique Medical, Tokyo, Japan), and a reduction of approximately 50% from baseline, indicative of MCAO, was confirmed in the rats. The rats were then allowed to recover from anesthesia at room temperature and were sacrificed after 24 h of reperfusion. All post-ischemic analyses (e.g., infarct volume, neurological scores, biochemical measurements, and gene expression) were performed 24 h after MCAO/Re (post-MCAO/Re), and the corresponding baseline measurements were obtained before MCAO/Re (pre-MCAO/Re). Rats that exhibited cerebral vessel bleeding after reperfusion were excluded from the experiment.

### 2.3. Neurological Evaluation

Post-ischemic neurological deficits were evaluated 24 h after reperfusion using a 5-point scale as follows: grade 0, no deficit; grade 1, failure to fully extend the right forepaw; grade 2, spontaneous circling or walking to the contralateral side; grade 3, walking only when stimulated; grade 4, unresponsiveness to stimulation with a depressed level of consciousness; and grade 5, death [12,13]. Before MCAO, all rats had a neurological score of zero. Rats that did not exhibit neurological deficits after MCAO/Re were excluded from the study. Neurological scores were assessed by an investigator blinded to the treatment protocol.

### 2.4. Infarct Assessment

After 24 h of reperfusion, the rats were anesthetized with isoflurane and decapitated. The brain was immediately removed and placed in ice-cold saline. Each brain was then sectioned into 2 mm coronal slices using a rat brain matrix. The slices were immediately immersed in 2% 2,3,5-triphenyl tetrazolium chloride (TTC, Fujifilm Wako Chemical Co.) at 37 °C for 15 min, followed by fixation in 4% formaldehyde [12,13]. Infarct areas were identified using an image analysis system (Scion Image 1.62, Frederick, MD, USA) and combined to calculate infarct volumes per brain using the following formula: corrected infarct volume (%) = (left hemisphere volume − (right hemisphere volume − the infarct volume)) × 100/left hemisphere volume.

### 2.5. Measurement of Total AA Level

The total AA (AA + DHA) levels in the cortex were determined using a spectrophotometric method with a Vitamin C Assay Kit (ROIK02, Shima Laboratories, Tokyo, Japan) [13], based on the 2,4-dinitrophenylhydrazine method [22]. Brain samples were collected at both time points: before MCAO/Re and 24 h after the procedure. Briefly, cortical tissue was homogenized in 5.4% metaphosphoric acid solution at a ratio of 1:14 (*w*/*v*). The homogenate was then centrifuged at 10,000× *g* for 15 min at 4 °C, and the supernatant was used for the assay.

### 2.6. Measurement of Lipid Peroxidation Level

The lipid peroxide content in brain tissue was determined using a spectrophotometric method with a thiobarbituric acid-reactive substances (TBARS) assay kit (Cayman Chemical, Ann Arbor, MI, USA), based on the TCA method [12,23]. Brain samples were collected at both time points: before MCAO/Re and 24 h after the procedure. Briefly, brain tissue (10% *w*/*v*) was homogenized in chilled RIPA buffer. The homogenate was then centrifuged at 1600× *g* for 10 min at 4 °C, and the supernatant was used for the assay.

### 2.7. Detection of O_2_^−^ Production in the Brain

Intracellular O_2_^−^ production in the ischemic penumbral region of the cortex before and after 24 h of MCAO/Re was detected by histochemical staining of freshly frozen brain sections (8 μm thick) using the fluorescent probe dihydroethidium (DHE) [13]. The brain sections were immediately incubated with DHE (10 μmol/L, Sigma-Aldrich Japan, Tokyo, Japan) in phosphate-buffered saline for 30 min at 37 °C. To assess the fluorescence intensity of oxidized DHE, three visual fields within the penumbral cortex of each hemisphere were photographed using an All-in-One fluorescence microscope (BZ-X700, Keyence, Osaka, Japan). Immunohistochemical analyses were conducted by an investigator blinded to the treatment protocol.

### 2.8. Detection of Cell Death in the Brain

Cell death in brain tissue was assessed using the DeadEnd™ Colorimetric TUNEL System kit (Promega, Madison, WI, USA), based on the terminal deoxyribonucleotidyl transferase-mediated biotin-16-dUTP nick-end labeling (TUNEL) procedure [23]. Coronal brain sections (8 μm thick) collected before MCAO/Re and 24W hours after the procedure were used for the assay. The slides were lightly counterstained with hematoxylin and observed under a microscope (BZ-X700, Keyence, Osaka, Japan). Quantification of TUNEL-positive cells was performed by counting cells in the ischemia-affected penumbral cortex. Three randomly selected visual fields were analyzed per region by an investigator blinded to the experimental conditions. The percentage of cell death was calculated using the apoptotic index, defined as the number of TUNEL-positive nuclei divided by the total number of nuclei.

### 2.9. Real-Time Polymerase Chain Reaction (PCR) Analysis

The expression levels of mRNA of pro-inflammatory cytokines (tumor necrosis factor-alpha (TNF-α) and interleukin-1 beta (IL-1β)); inflammatory mediators (cyclooxygenase-2 (COX-2) and matrix metalloproteinase-9 (MMP-9)); oxidative mediators (heme oxygenase-1 (HO-1)); and antioxidant enzymes (superoxide dismutase (SOD), catalase, and glutathione peroxidase (GPx)) were assessed by quantitative real-time polymerase chain reaction (PCR), as described previously [13,24]. Rats subjected to MCAO were sacrificed after 24 h of reperfusion, and total RNA was extracted from the ischemic penumbral cortex of each rat using the RNeasy Mini Kit (QIAGEN, Hilden, Germany) following the manufacturer’s protocol. Total RNA was also extracted from brain samples collected before MCAO/Re. Total RNA (500 pg per sample) was reverse-transcribed using oligo-dT and random hexamer primers with PrimeScript RT Enzyme Mix I (Takara RNA PCR Kit, Takara Biomedicals, Shiga, Japan). Real-time PCR was performed using 10 ng of cDNA and a pair of gene-specific primers (Takara Biomedicals) in SYBR Premix Ex Taq (Takara Biomedicals). Amplification was conducted on an iCycler iQ Real-Time Detection System (Bio-Rad Laboratories, Hercules, CA, USA) under the following conditions: one cycle at 95 °C for 10 s, followed by 50 cycles at 95 °C for 5 s and 60 °C for 34 s. β-Actin expression was used as an internal control to normalize cDNA levels. The specificity of the PCR amplification was confirmed by melting curve analysis. Data were expressed as the mean ± SD relative to the pre-MCAO/Re 0% AA group.

### 2.10. Statistical Analysis

One-way ANOVA followed by post hoc Tukey’s multiple-comparisons test was used for statistical analysis. Neurological deficit scores were analyzed using the Kruskal–Wallis test, followed by the Mann–Whitney U test. In all cases, a *p* value of <0.05 was assumed to denote statistical significance.

Outlier analysis was performed using the ROUT method (Q = 1%), and identified outliers were excluded from analysis. In addition, animals that died within 24 h after MCAO/Re or plasma samples with visible hemolysis were excluded from the respective analyses.

## 3. Results

### 3.1. Body Weight, Food Intake, and Water Intake

To accurately evaluate the effects of AA deficiency, it is crucial to consider the physiological characteristics of experimental animals when applying these findings to human physiology. Like humans and guinea pigs, ODS rats lack the ability to synthesize AA endogenously, making them a suitable model for studying AA deficiency.

After housing each ODS rat group (0%, 0.1%, and 1% AA) on an AA-deficient diet for two weeks, we measured their body weight, food intake, and water intake (Table 1). Although some cases of anorexia were observed in the 0% AA group, there was no significant weight loss or visible physical abnormalities, suggesting that the rats were in the early stages of AA deficiency. The daily AA intake in the 0.1% AA group was adjusted to approximately 40 mg per rat, based on feed consumption and biosynthesis levels in normal rats. In contrast, the 1% AA group had unrestricted access to AA, resulting in a daily intake of approximately 450 mg per rat, which is considered a high intake level. No significant differences in body weight were observed among the groups. While food consumption increased with AA supplementation, water intake remained unchanged. Additionally, no physical abnormalities were observed in any group.

### 3.2. Ischemic Brain Injury and Neurological Deficits

**Figure 1:**
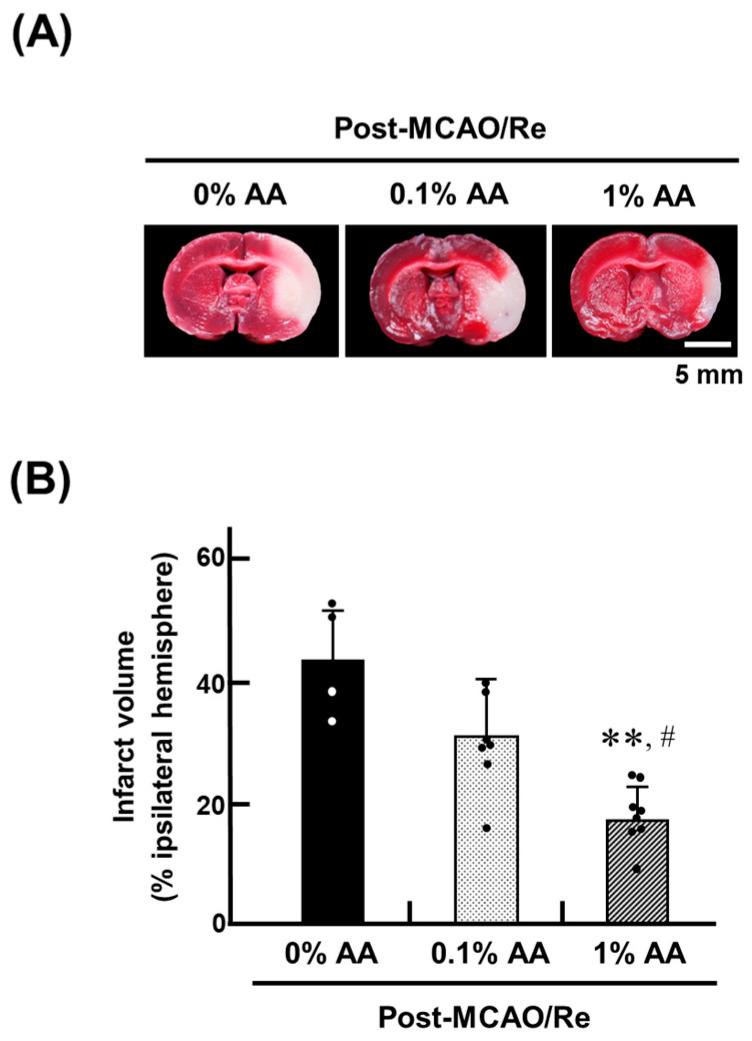
Effects of AA deficiency and supplementation on middle cerebral artery occlusion and reperfusion (MCAO/Re)-induced infarction in ODS rat brains. (**A**) Representative 2,3,5-triphenyl tetrazolium chloride (TTC)-stained coronal brain sections from ODS rats fed an AA-free diet (0%) or supplemented with the 0.1% or 1% AA for two weeks before MCAO/Re. (**B**) Infarct volume in the ischemic hemispheres of ODS rats, assessed by TTC staining 24 h after MCAO/Re (post-MCAO/Re). Data are presented as the mean ± SD (n = 4–8 per group). ** *p* < 0.01 vs. 0% AA group; ^#^ *p* < 0.05 vs. 0.1% AA group.

After MCAO/Re, the infarct volume was assessed 24 h later using TTC staining of cerebral coronal sections (Figure 1). The 0% AA group exhibited a severe infarct extending to a large part of the left striatum and cortex. In contrast, the 1% AA group showed a significant reduction in infarct size, suggesting that AA supplementation mitigates ischemic damage. The 0.1% AA group also exhibited a trend toward decreased infarct volume compared with the 0% AA group, although this reduction was not statistically significant. Compared with the 0% AA group, infarct volume was reduced by approximately 24% in the 1% AA group, where infarction was primarily confined to the cortex and parts of the striatum.

**Figure 2:**
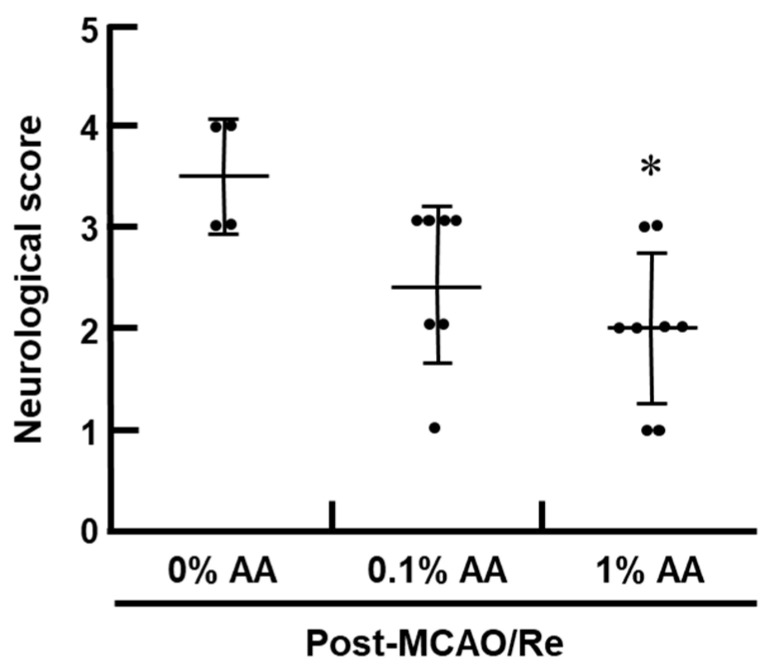
Effects of AA deficiency and supplementation on MCAO/Re-induced neurological deficits in ODS rats. Neurological deficits were assessed 24 h after MCAO/Re using a 5-point scale. Data are presented as the mean ± SD (n = 4–8 per group). * *p* < 0.05 vs. 0% AA group.

Neurological deficits were assessed 24 h after MCAO/Re (Figure 2). Consistent with the infarct volume results, the 0% AA group exhibited the most severe neurological deficits, indicating that AA deficiency exacerbates functional impairment following ischemic injury. In contrast, the 1% AA group showed a significant reduction in neurological deficits, suggesting that AA supplementation mitigates the severity of ischemic damage. The 0.1% AA group also exhibited a trend toward improved neurological function compared with the 0% AA group, although this improvement was not statistically significant.

### 3.3. Total AA Levels in the Cortex

**Figure 3:**
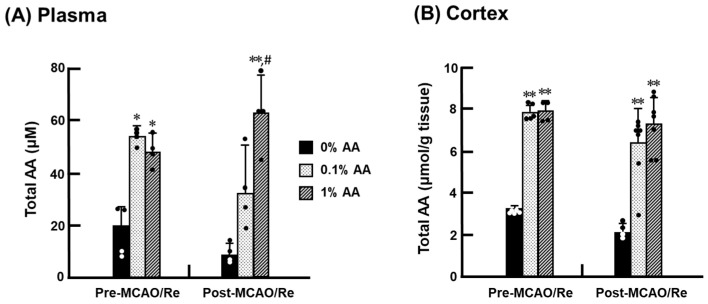
Effects of AA deficiency and supplementation on total AA (AA + dehydroascorbic acid; DHA) levels in the plasma (**A**) and cortex (**B**) of ODS rats. Total AA (AA + DHA) levels in the cortex were measured in ODS rats pre- and post-MCAO/Re. Data are presented as the mean ± SD (n = 4–7). *^,^ ** *p* < 0.05, 0.01 vs. 0% AA group at pre- or post-MCAO/Re, respectively; ^#^ *p* < 0.05 vs. 0.1% AA group.

The total content of AA (oxidized + reduced form) in the plasma and cerebral cortex of ODS rats was measured (Figure 3). DHA has been demonstrated to be rapidly reduced to AA or decomposed into 2,3-diketo-1-gulonic acid in the brain, resulting in DHA concentrations ranging from 0% to 2% of total AA levels [13]. Therefore, the total AA levels can be considered nearly equivalent to the AA levels. Before MCAO/Re, the total AA levels were low in both the plasma (19.9 ± 7.8 μM) and cerebral cortex (9.7 ± 0.2 μmol/g) of the 0% AA group. AA supplementation increased the total AA levels, with no significant difference between the 0.1% and 1% AA groups. Following MCAO/Re, the 0% AA group showed a trend toward decreased AA levels compared with its pre-MCAO/Re level, suggesting that AA deficiency exacerbates AA depletion following ischemic stress. In contrast, the AA levels in the 1% AA group were maintained, showing no significant difference from the pre-MCAO/Re levels. The 0.1% AA group showed a trend toward decreased total AA levels, especially in the plasma after MCAO/Re, but this reduction was not statistically significant.

### 3.4. Lipid Peroxidation Levels in the Cortex

**Figure 4:**
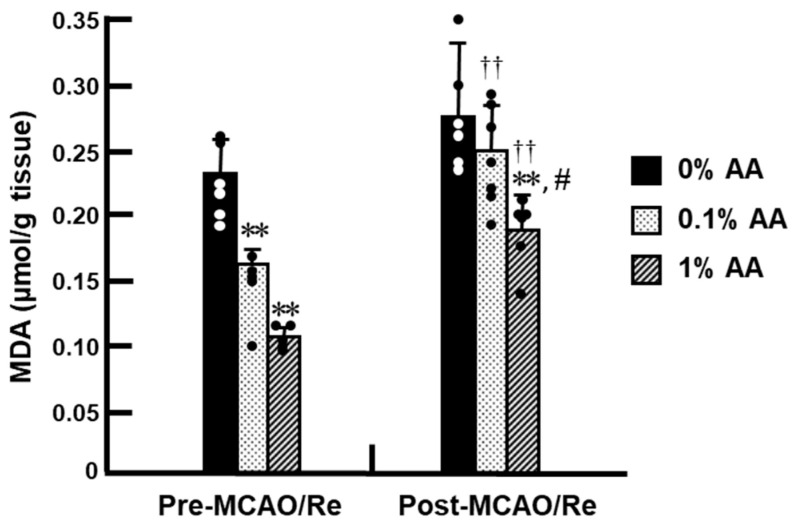
Effects of AA deficiency and supplementation on lipid peroxidation levels in the brain cortex of ODS rats pre- and post-MCAO/Re. Lipid peroxidation levels were assessed using the thiobarbituric acid-reactive substances (TBARS) assay in the brain cortex of ODS rats pre- and post-MCAO/Re. Data are presented as the mean ± SD (n = 5–7). ** *p* < 0.01 vs. 0% AA group at pre- or post-MCAO/Re, respectively; ^#^ *p* < 0.05 vs. 0.1% AA group; ^††^ *p* < 0.01 vs. the corresponding group at pre-MCAO/Re.

Before MCAO/Re, lipid peroxidation levels were significantly higher in the 0% AA group compared with the 0.1% and 1% AA groups, indicating that AA deficiency promotes oxidative stress in the cortex (Figure 4). In contrast, both the 0.1% and 1% AA groups showed a significant reduction in lipid peroxidation compared with the 0% AA group, with a greater reduction observed in the 1% AA group, indicating a dose-dependent decrease. Following MCAO/Re, lipid peroxidation levels increased in all groups compared with pre-MCAO/Re levels. The 0% AA group exhibited a moderate increase (~1.2-fold compared with pre-MCAO/Re levels). Notably, the 0.1% AA group also showed a significant increase in MDA levels, reaching values comparable to those in the 0% AA group, indicating that 0.1% AA supplementation did not effectively prevent lipid peroxidation after MCAO/Re. In contrast, the 1% AA group exhibited only a minimal increase (~1.1-fold), maintaining significantly lower lipid peroxide levels compared with the other groups.

### 3.5. O_2_^−^ Production After Ischemia with Reperfusion

**Figure 5:**
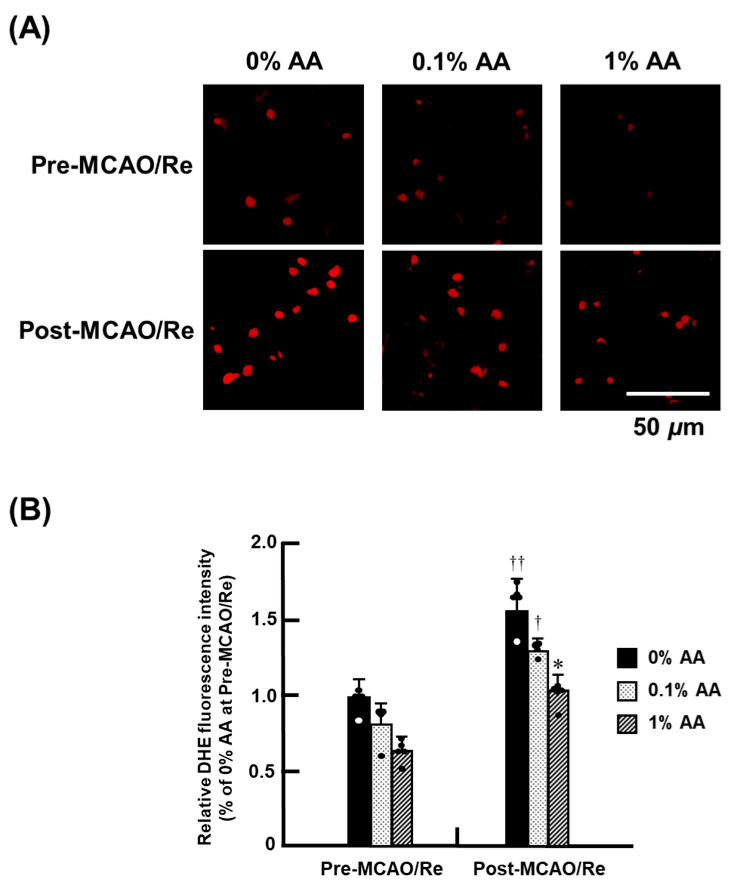
Effects of AA deficiency and supplementation on superoxide (O_2_^−^) production in the penumbral cortex of ODS rats pre- and post-MCAO/Re. (**A**) Representative images of superoxide production detected by dihydroethidium (DHE) staining in coronal cortex sections from ODS rats. (**B**) Quantitative analysis of DHE fluorescence intensity in the cortex. Data are presented as the mean ± SD (n = 5). * *p* < 0.05 vs. 0% AA group; ^†, ††^ *p* < 0.05, 0.01 vs. the corresponding group at pre-MCAO/Re.

The production of O_2_^−^ was assessed based on the fluorescence intensity of brain tissue sections using DHE staining (Figure 5). Representative histological images of DHE staining for the 0% AA, 0.1% AA, and 1% AA groups are shown in Figure 5A, and fluorescence intensity is quantified in Figure 5B. Before MCAO/Re, DHE-positive cells were sparsely distributed in the cortex of all groups, with a tendency for fewer cells in the 1% AA group compared to the 0% AA group. Following MCAO/Re, DHE-positive cells increased in all groups, with the most pronounced increase observed in the 0% AA group. In contrast, the 1% AA group exhibited a significantly lower increase in DHE-positive cells in the penumbral cortex compared with the 0% AA group, suggesting that higher AA levels mitigate ischemia-induced O_2_^−^ production. The 0.1% AA group showed no significant difference from the 0% AA group, indicating that 0.1% AA supplementation was insufficient to suppress ischemia-induced superoxide production.

### 3.6. Cell Death Induced by Ischemia with Reperfusion

**Figure 6:**
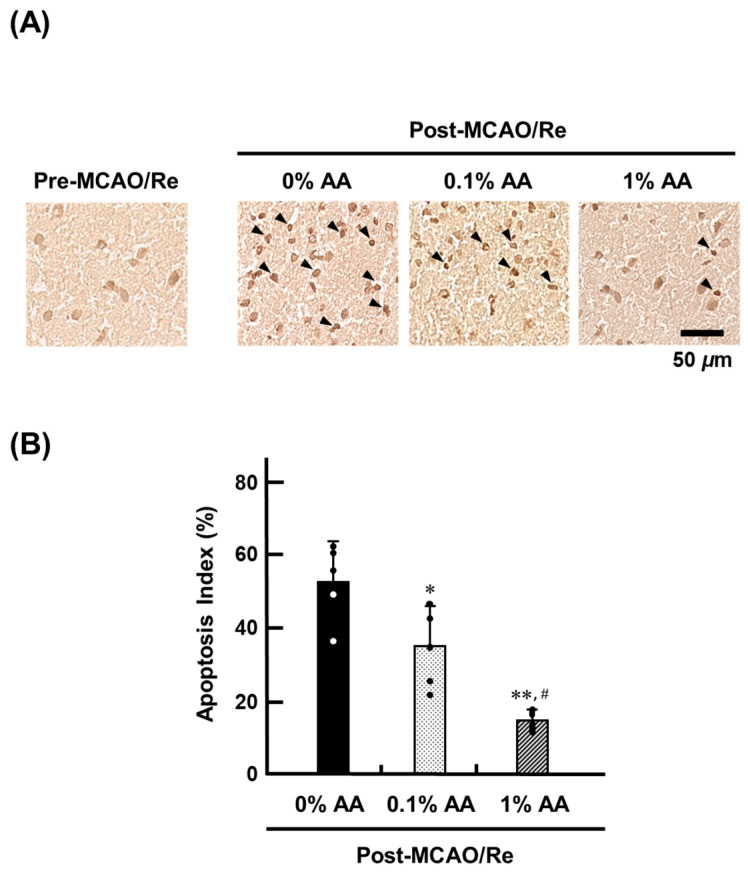
Effects of AA deficiency and supplementation on neuronal apoptosis induced by MCAO/Re in the penumbral cortex of ODS rats. (**A**) Representative images of apoptotic cells detected by terminal deoxyribonucleotidyl transferase-mediated dUTP nick-end labeling (TUNEL) staining in the penumbral cortex post-MCAO/Re. (**B**) Quantitative analysis of TUNEL-positive cell index. Data are presented as the mean ± SD (n = 5). *^,^ ** *p* < 0.05, 0.01 vs. 0% AA group; ^#^ *p* < 0.05 vs. 0.1% AA group.

The extent of nucleosome DNA fragmentation in apoptotic cell death in the penumbra region of the cerebral cortex after MCAO/Re was evaluated using TUNEL staining (Figure 6). Before MCAO/Re, no TUNEL-positive cells were observed in any group. However, following MCAO/Re, TUNEL-positive cells were detected in all groups, with the most pronounced increase in the 0% AA group. In contrast, the number of TUNEL-positive cells was significantly reduced in the 0.1% AA group compared with the 0% AA group, showing 51.7% reduction, indicating that standard-dose AA supplementation provides moderate neuroprotection against ischemia-induced apoptosis. The 1% AA group exhibited an even greater reduction in TUNEL-positive cells, supporting a dose-dependent protective effect of AA supplementation.

### 3.7. Alterations in the Expression of Inflammatory and Antioxidant Genes in the Penumbral Cortex

**Figure 7:**
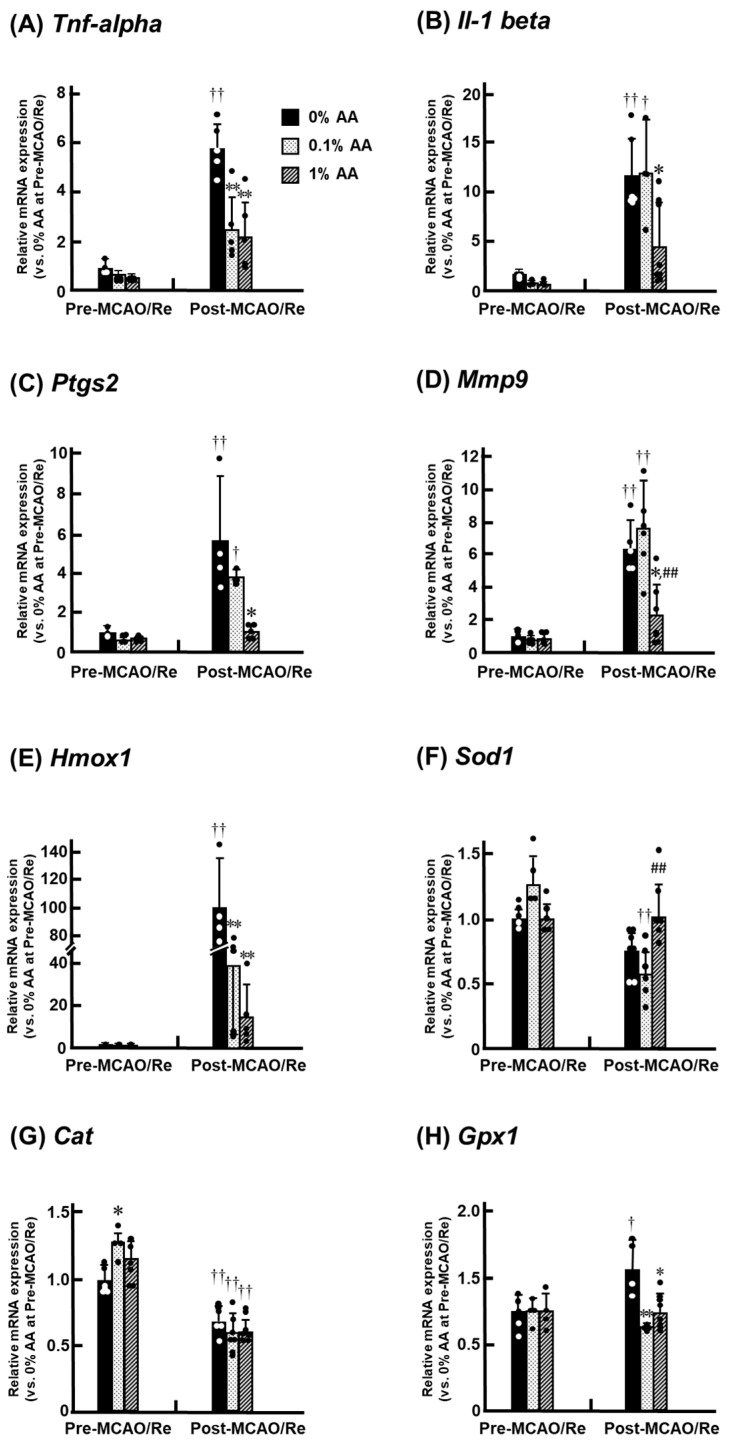
Effects of AA deficiency and supplementation on mRNA expression of pro-inflammatory cytokines and antioxidant enzymes in the penumbral cortex pre- and post-MCAO/Re. Gene expression levels of tumor necrosis factor-alpha (TNF-α) (**A**), interleukin-1 beta (IL-1β) (**B**), cyclooxygenase-2 (COX-2; *Ptgs2*) (**C**), matrix metalloproteinase-9 (MMP-9) (**D**), heme oxygenase-1 (HO-1) (**E**), superoxide dismutase (SOD) (**F**), catalase (Cat) (**G**), and glutathione peroxidase (GPx) (**H**) were determined by real-time PCR in the penumbral cortex of ODS rats. Data are presented as the mean ± SD (n = 4–7). *^,^ ** *p* < 0.05, 0.01 vs. 0% AA group at pre- or post-MCAO/Re, respectively; ^##^ *p* < 0.01 vs. 0.1% AA group; ^†, ††^ *p* < 0.05, 0.01 vs. the corresponding group at pre-MCAO/Re.

Before MCAO/Re, the mRNA expression levels of IL-1β (*Il-1 beta*) and COX-2 (*Ptgs2*) were slightly higher in the 0% AA group compared to the AA-supplemented groups, suggesting that AA deficiency may promote a pro-inflammatory state (Figure 7). Following MCAO/Re, the inflammation-related gene expression significantly increased in all groups compared with pre-MCAO/Re levels. In the 0% AA group, TNF-α mRNA expression increased approximately 6-fold, IL-1β about 12-fold, COX-2 about 6-fold, and MMP-9 about 6-fold.

Conversely, in the 1% AA group, these increases were significantly suppressed, suggesting a protective effect of high-dose AA supplementation against ischemia-induced inflammation. The mRNA expression of HO-1 (Hmox1) increased substantially (~100-fold) in the 0% AA group after MCAO/Re, whereas the increase was relatively moderate (~15-fold) in the 1% AA group. SOD mRNA (*Sod1*) and catalase mRNA (*Cat*) expression levels before MCAO/Re were comparable among the groups, whereas catalase mRNA expression was significantly higher in the 0.1% AA group compared with the 0% AA group. Following MCAO/Re, the SOD and catalase mRNA expression levels decreased in all groups. However, in the 1% AA group, only the decline in SOD mRNA expression was mitigated, while catalase expression remained unaffected. Furthermore, GPx mRNA (*Gpx1*) expression increased after MCAO/Re in the 0% AA group, whereas in the AA-supplemented groups (0.1% and 1%), GPx mRNA expression remained the pre-MCAO/Re levels.

### 3.8. Alterations in the Expression of SVCT2 and GLUT1 mRNA in the Penumbra Cortex

**Figure 8:**
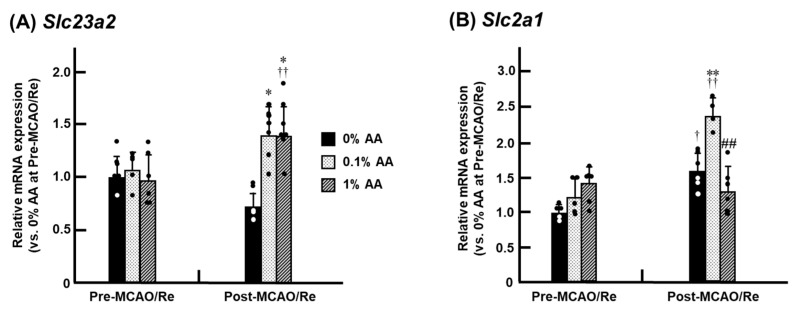
Effects of AA supplementation on sodium-dependent vitamin C transporter 2 (SVCT2) and glucose transporter 1 (GLUT1) mRNA expression in the ischemic penumbral cortex of ODS rats. The expression levels of SVCT2 mRNA (*Slc23a2*) (**A**) and GLUT1 mRNA (*Slc2a1*) (**B**) were determined by real-time PCR in the penumbral cortex of ODS rats pre- and post-MCAO/Re. Data are presented as the mean ± SD (n = 5–7). *^,^ ** *p* < 0.05, 0.01 vs. 0% AA group at pre- or post-MCAO/Re, respectively; ^##^ *p* < 0.01 vs. 0.1% AA group; ^†, ††^ *p* < 0.05, 0.01 vs. the corresponding group at pre-MCAO/Re.

The mRNA expression of AA transporters, SVCT2 (*Slc23a2*) and GLUT1 (*Slc2a1*), was analyzed in the cerebral cortex (Figure 8). Before MCAO/Re, no alteration was observed in SVCT2 mRNA expression in the three experimental groups. However, following MCAO/Re, SVCT2 mRNA expression increased by approximately 1.5-fold in the 0.1% and 1% AA groups compared with pre-MCAO/Re levels. In contrast, no significant increase was observed in the 0% AA group, suggesting that AA deficiency impairs ischemia-induced upregulation of SVCT2 mRNA expression (Figure 8A). For GLUT1 mRNA expression, MCAO/Re induced an increase in the 0% and 0.1% AA groups. Notably, in the 0.1% AA group, GLUT1 mRNA expression exhibited a significant increase of approximately 1.8-fold compared with pre-MCAO/Re levels. However, in the 1% AA group, no alteration in the expression of GLUT1 mRNA was observed, regardless of MCAO/Re (Figure 8B).

## 4. Discussion

In this study, we investigated the effects of AA deficiency on cerebral damage following transient cerebral ischemia in ODS rats. ODS rats, which lack endogenous AA synthesis, serve as a useful model for replicating human AA deficiency and evaluating the isolated protective effects of AA in ischemic brain injury, unlike normal rats that can synthesize AA endogenously. Before MCAO/Re, the 0% AA group exhibited a significant reduction in the plasma and intracerebral AA levels compared to the AA-supplemented groups, both of which were mitigated by supplementation with either 0.1% or 1% AA (Figure 3 and Figure 4). Brain damage induced by MCAO/Re, including infarct formation and neurological symptoms, was more pronounced in the 0% AA group than in the AA-supplemented groups (Figure 1 and Figure 2). In addition, increased lipid peroxidation (Figure 4), O_2_^−^ production (Figure 5), and inflammation-related gene expression (Figure 7) possibly contributed to neuronal cell death (Figure 6). In contrast, the 1% AA group exhibited reduced lipid peroxidation, O_2_^−^ production, COX-2 and MMP-9 expressions, and fewer TUNEL-positive cells, suggesting that the reduction in infarct volume and neuronal deficits are attributed to the suppression of cell death. These results suggest that sufficient AA intake mitigates oxidative damage in the brain, suppresses inflammatory responses, and prevents neuronal cell death associated with ischemic brain injury.

The brain consumes approximately 20% of the body’s total oxygen, making it highly susceptible to ROS generation. Mitochondria are a primary source of ROS production and are subjected to increasing oxidative stress while generating energy. AA helps maintain mitochondrial membrane potential and reduce O_2_^−^ production, especially in damaged cells with electron transport chain deficiencies [25,26]. The central nervous system contains relatively high levels of AA, which is rapidly utilized in the early stages of ROS elimination and works synergistically with other antioxidants, such as glutathione (GSH), coenzyme Q10, and vitamin E (VE), to protect against oxidative damage [27,28]. Even under AA deficiency, the brain maintains higher AA concentrations than other organs to preserve its neuroprotective function [21,29]. Hughes et al. reported that intracerebral AA concentrations remained at approximately 25% of normal levels even when undetectable in other organs of guinea pigs with scurvy [30]. Consistent with these findings, our study shows that AA-deprived rats retained about 40% of the intracerebral AA levels observed in AA-supplemented groups. Despite excessive AA intake in the 1% AA group, intracerebral AA levels were comparable to those in the 0.1% AA group (Figure 3). This is likely due to AA being water soluble and not storable beyond a certain threshold. A previous study demonstrated that even when ODS rats were fed a diet containing 800 mg/kg AA for 15 days, AA concentrations in the liver and plasma remained comparable to those in normal rats fed an AA-free diet [31].

Oxidative stress plays a key role in the development and progression of damage during transient cerebral ischemia. Notably, reperfusion following prolonged vascular occlusion can trigger a surge in ROS production, leading to cellular damage and, ultimately, apoptosis induction [10,11,13]. In this study, we subjected AA-deprived rats to MCAO/Re and examined the impact of AA intake on brain tissue. Transient cerebral ischemia led to a reduction in AA levels in the cerebral cortex across all groups, accompanied by increased O_2_^−^ production and lipid peroxidation (Figure 4 and Figure 5). Under AA-deficient conditions, the infarct volume significantly increased, along with a worsening of neurological symptoms. Conversely, AA supplementation reduced the infarct volume by approximately 50% in the 1% AA group compared to the 0% AA group, demonstrating its role in mitigating ischemic damage by reducing oxidative stress and preserving neuronal integrity. Interestingly, the intracerebral AA levels did not significantly differ between the 0.1% and 1% AA groups. Nonetheless, oxidative damage was significantly reduced, and ischemic cerebral injury was markedly alleviated only in the 1% AA group, suggesting that a high level of AA intake plays a crucial role in neuroprotection (Figure 1 and Figure 2). Beyond direct ROS scavenging, higher AA intake may also support other antioxidant systems, such as VE and GSH regeneration [1,21].

Inflammatory neurodegeneration is another critical process contributing to brain damage following ischemia and reperfusion. ROS activate NF-κB, a key regulator of inflammation-related gene expression, including TNF-α, IL-1β, and COX-2, which further amplifies the inflammatory response. The upregulation of TNF-α and IL-1β enhances MMP expression, leading to BBB disruption and exacerbating ischemic injury [32]. Our findings demonstrate that while inflammation-related gene expression in the cerebral cortex remained unchanged in the early stages of AA deficiency, ischemia and reperfusion markedly upregulated these genes in the penumbra, triggering a pronounced inflammatory response. Notably, MCAO/Re-induced upregulation of TNF-α, IL-1β, and COX-2 mRNA expression was suppressed in the 1% AA group but not in the 0.1% AA group (Figure 7B–D). Similarly, Kawade et al. reported that AA deficiency in ODS rats triggers inflammation in the intestine and liver by exacerbating oxidative stress and that only sufficient AA supplementation effectively prevents inflammation [33,34]. Previous studies suggest that COX inhibitors significantly suppress MMP-mediated BBB disruption during neuroinflammation [35], and MMP inhibitors mitigate ischemic brain injury [36]. Additionally, intracellular AA has been shown to inhibit nuclear factor kappa-B (NF-κB) activation, thereby preventing BBB dysfunction. Thus, the anti-inflammatory effects of high AA intake may act upstream in the inflammatory cascade and, in combination with its antioxidant properties, contribute to cerebroprotection. One limitation of the present study is the absence of protein-level validation. Nevertheless, mRNA analysis remains a widely accepted approach for capturing early transcriptional responses, especially for genes related to cytokine signaling and oxidative stress, which are often transient and dynamically regulated. In our previous studies, changes in mRNA expression were found to parallel protein-level alterations, supporting the biological relevance of transcript-based findings under similar ischemic conditions [37].

In this study, we investigated the effects of AA deficiency and AA supplementation on HO-1 mRNA expression. Our results demonstrated that ischemia upregulated HO-1 mRNA expression in all groups (Figure 7E). Several experimental studies have demonstrated that moderate induction of HO-1 reduces oxidative stress, suppresses pro-inflammatory cytokines, and inhibits neuronal apoptosis in the brain [38,39]. However, excessive HO-1 mRNA expression has been reported to exacerbate neuronal damage by promoting the accumulation of free iron, which enhances oxidative stress through the Fenton reaction [40]. Notably, in the 0% AA group, ischemic stress increased HO-1 mRNA expression by more than 100-fold compared to pre-ischemic levels, whereas almost no alteration was observed in its expression level in 1% AA group in comparison to that of pre-MCAO/Re.

These findings suggest that a high level of AA intake mitigates MCAO/Re-induced brain injury by optimally regulating HO-1 mRNA expression. To prevent oxidative stress, AA activates the antioxidant system to eliminate free radicals, not only at the level of low molecular weight antioxidants, such as GSH, coenzyme Q10, and VE, but also by modulating antioxidant enzyme activity [1]. We found that MCAO/Re led to a decrease in SOD mRNA expression; however, this reduction was mitigated in the 1% AA group. These findings suggest that sufficient AA intake not only suppresses O_2_^−^ production in brain tissue but also prevents the ischemia-induced decline in SOD expression, which may help reduce ROS formation from the early stages of ischemic stress. Meanwhile, catalase expression was significantly reduced in all groups following ischemic stress, and this reduction was not mitigated by AA supplementation. Since catalase expression in brain tissue is lower than in other organs, GPx plays a particularly important role in decomposing H_2_O_2_ during ischemia/reperfusion [41,42]. Following ischemia, GPx mRNA expression increased in the 0% AA group but remained at pre-ischemic levels in the AA-supplemented groups (Figure 7H). Wolf et al. reported that GSH levels increase in the hippocampus of juvenile ODS rats, contributing to neuroprotection [43]. AA not only acts as a direct antioxidant but also as a substrate for ascorbate peroxidase, an enzyme that detoxifies H_2_O_2_ and other peroxides. In ODS rats, AA deficiency may specifically enhance the glutathione system as a compensatory response.

Effective transport of AA to the brain is essential for capturing and eliminating ROS. The well-known cerebral AA transporters include SVCT2, which is primarily expressed in the choroid plexus and neurons and is responsible for transporting reduced AA, as well as GLUT1, which facilitates the transport of DHA [21]. We measured the expression levels of these transporter genes in the cerebral cortex and found no significant differences in SVCT2 mRNA expression among the groups before MCAO/Re (Figure 8). This finding aligns with a previous study reporting that AA deficiency does not affect SVCT2 expression at either the genetic or protein level in the brain, including the hippocampus, cerebellum, and cortex [5]. MCAO/Re significantly increased SVCT2 expression in the 0.1% and 1% AA groups, suggesting that ischemic stress may promote AA utilization via SVCT2 (Figure 8A). This observation is further supported by a previous report using a mouse MCAO model, in which SVCT2 protein expression was elevated by approximately 1.5-fold at 24 h and increased further at 5 days post-ischemia, in parallel with enhanced AA uptake [20]. Although interspecies differences must be considered, these findings imply that the observed mRNA upregulation may represent an early biologically meaningful response that precedes functional protein-level changes. In contrast, no such increase in SVCT2 expression was observed in the 0% AA group. Seno et al. reported that cytokines such as TNF-α and IL-1 suppress AA transport via SVCT2 in human umbilical vein endothelial cells [44]. It is therefore plausible that the reduced SVCT2 expression in the 0% AA group may be partially attributable to the upregulation of these pro-inflammatory cytokines. GLUT1 mRNA expression increased in the 0% and 0.1% AA groups but remained unchanged in the 1% AA group (Figure 8B). During ischemia-reperfusion, increased energy demand and oxidative stress have been reported to alter GLUT1 mRNA expression and function [12,13,21]. Under ischemic conditions, elevated oxidative stress converts AA into DHA, making GLUT1-mediated DHA uptake essential for maintaining intracellular AA levels. Thus, MCAO/Re may have exacerbated oxidative stress in the brains of rats with insufficient AA, inducing a compensatory upregulation of GLUT1 mRNA expression. One possible explanation for the lack of change in GLUT1 mRNA expression in the 1% AA group following MCAO/Re is that high AA intake effectively suppressed oxidative stress, reducing the conversion of AA to DHA and eliminating the need for a compensatory increase in GLUT1 expression. This study focused on the early transcriptional responses of AA transporters and other genes involved in ischemic brain injury. Although differences were observed among the experimental groups, the extent to which AA intake modulates transporter expression remains to be clarified. Direct functional assays—such as transporter inhibition or DHA/AA uptake measurements—were not feasible within this experimental design. Such analyses typically require alternative systems, such as cultured cells or genetically modified models, where transporter activity and dosing conditions can be precisely controlled [45,46,47]. Furthermore, clarification of the apparent threshold between 0.1% and 1% AA supplementation may benefit from future studies addressing saturation kinetics or pharmacokinetic behavior. In future work, these approaches will be essential to further elucidate the mechanistic contribution of SVCT2 and GLUT1 to the neuroprotective actions of AA. Although BBB disruption may allow AA to passively enter the brain parenchyma following ischemia/reperfusion, it remains unclear whether this contributes to neuroprotection, as regions with significant BBB breakdown are typically associated with severe and often irreversible tissue damage. Therefore, the contribution of AA transported across a disrupted BBB to functional recovery may be limited. Further studies would be needed to clarify the interplay between BBB integrity, AA transport, and neuroprotective efficacy.

While the present findings demonstrate the acute neuroprotective effects of AA following ischemia, it remains to be determined whether these effects are sustained over time. Ischemic brain injury progresses beyond the acute phase, involving delayed neuronal damage and the activation of repair mechanisms such as inflammation resolution, neurogenesis, and tissue remodeling [48]. Therefore, further studies are warranted to explore the long-term efficacy of AA supplementation and its potential role in promoting recovery during the subacute and chronic phases of stroke.

## 5. Conclusions

The present study demonstrated that AA deficiency exacerbates ischemic brain injury by increasing oxidative stress and neuroinflammation. In ODS rats, AA depletion resulted in elevated lipid peroxidation, O_2_^−^ production, and the upregulation of pro-inflammatory cytokines, leading to more severe ischemic damage. While low-dose AA supplementation (0.1%) was insufficient to mitigate this damage, high-dose AA (1%) effectively suppressed oxidative stress, downregulated COX-2 and MMP-9 mRNA expression, and decreased infarct size. Furthermore, the upregulation of SVCT2 in AA-supplemented groups suggests enhanced AA transport under ischemic conditions. These findings suggest the critical role of adequate AA levels in protecting cerebrovascular integrity and reducing ischemic injury.

## Figures and Tables

**Table 1:** Effects of supplementation with L-ascorbic acid (AA) on body weight, food intake, and water intake in osteogenic disorder Shionogi (ODS) rat groups.

Groups	Body Weight (g)	Food Intake (g/Day)	Water Intake (mL/Day)	EstimatedAA Intake (mg)
0% AA	244.8 ± 9.8	16.1 ± 3.1	34.0 ± 7.6	―
0.1% AA	259.1 ± 8.9	21.8 ± 2.4 **	36.2 ± 8.1	36.2 ± 8.1
1% AA	242.1 ± 18.0	20.5 ± 2.1 **	46.5 ± 12.8	465 ± 128

The data show the mean ± SD. ** *p* < 0.01 vs. the 0% AA groups (n = 6–8). ODS rats were given free access to AA-deficient rodent chow.

## Data Availability

All data supporting the findings of this study are included within the article. No additional datasets were generated or analyzed during the current study.

## Abbreviations

AA	L-ascorbic acid
BBB	blood–brain barrier
CAT	catalase
CNS	central nervous system
COX-2	cyclooxygenase-2
DHA	dehydroascorbic acid
DHE	dihydroethidium
GLUT1	glucose transporter 1
GLO	L-gulonolactone oxidase
GPx	glutathione peroxidase
GSH	glutathione
HO-1	heme oxygenase-1
H_2_O_2_	hydrogen peroxide
IL-1β	interleukin-1 beta
MCAO/Re	middle cerebral artery occlusion and reperfusion
MDA	malondialdehyde
MMP-9	matrix metalloproteinase-9
NF-κB	nuclear factor kappa-B
O_2_^−^	superoxide
ODS rat	osteogenic disorder Shionogi rat
PCR	quantitative real-time polymerase chain reaction
ROS	reactive oxygen species
SOD	superoxide dismutase
SVCT2	sodium-dependent vitamin C transporter 2
TBARS	thiobarbituric acid-reactive substances
TNF-α	tumor necrosis factor-alpha
TTC	2,3,5-triphenyl tetrazolium chloride
TUNEL	terminal deoxyribonucleotidyl transferase-mediated biotin-16-dUTP nick-end labeling
VE	vitamin E
